# Risk prediction for coronary heart disease by a genetic risk score - results from the Heinz Nixdorf Recall study

**DOI:** 10.1186/s12881-020-01113-y

**Published:** 2020-09-10

**Authors:** Sonali Pechlivanis, Nils Lehmann, Per Hoffmann, Markus M. Nöthen, Karl-Heinz Jöckel, Raimund Erbel, Susanne Moebus

**Affiliations:** 1 Institute for Medical Informatics, Biometry and Epidemiology, University Hospital of Essen, University Duisburg-Essen, Essen, Germany; 2 Department of Genomics, Life & Brain Center, University of Bonn, Bonn, Germany; 3 Division of Medical Genetics, Department of Biomedicine, University of Basel, Basel, Switzerland; 4 Centre for Urban Epidemiology, University Hospital Essen, Essen, Germany

**Keywords:** Coronary heart disease, Coronary artery disease, Coronary artery calcification, Genetic risk score, Cohort study

## Abstract

**Background:**

A Genetic risk score for coronary artery disease (CAD) improves the ability of predicting coronary heart disease (CHD). It is unclear whether i) the use of a CAD genetic risk score is superior to the measurement of coronary artery calcification (CAC) for CHD risk assessment and ii) the CHD risk assessment using a CAD genetic risk score differs between men and women.

**Methods:**

We included 4041 participants (age-range: 45–76 years, 1919 men) of the Heinz Nixdorf Recall study without CHD or stroke at baseline. A standardized weighted CAD genetic risk score was constructed using 70 known genetic variants. The risk score was divided into quintiles (Q1-Q5). We specified low (Q1), intermediate (Q2-Q4) and high (Q5) genetic risk groups. Incident CHD was defined as fatal and non-fatal myocardial infarction, stroke and coronary death. The association between the genetic risk score and genetic risk groups with incident CHD was assessed using Cox models to estimate hazard ratios (HR) and 95%-confidence intervals (CI). The models were adjusted by age and sex (Model1), as well as by established CHD risk factors (RF) and CAC (Model2). The analyses were further stratified by sex and controlled for multiple testing.

**Results:**

During a median follow-up time of 11.6 ± 3.7 years, 343 participants experienced CHD events (219 men). Per-standard deviation (SD) increase in the genetic risk score was associated with 18% increased risk for incident CHD (Model1: *p* = 0.002) which did not change after full adjustment (Model2: HR = 1.18 per-SD (*p* = 0.003)). In Model2 we observed a 60% increased CHD risk in the high (*p* = 0.009) compared to the low genetic risk group. Stratifying by sex, only men showed statistically significantly higher risk for CHD (Model2: HR = 1.23 per-SD (*p* = 0.004); intermediate: HR = 1.52 (*p* = 0.04) and high: HR = 1.88 (*p* = 0.008)) with no statistically significant risk observed in women.

**Conclusion:**

Our results suggest that the CAD genetic risk score could be useful for CHD risk prediction, at least in men belonging to the higher genetic risk group, but it does not outbalance the value of CT-based quantification of CAC which works independently on both men and women and allows better risk stratification in both the genders.

## Background

Coronary heart disease (CHD) is one of the leading causes of deaths with over 1.8 million and 836,546 deaths in European Union as well as in the USA respectively, at the estimated annual cost of €210 billion in the European Union and $329.7 billion in the USA [1, 2]. Within the European Union, CHD is the second cause of death with around 24% death among men and 17% among women under 65 years [1]. The European Association for Cardiovascular Prevention and Rehabilitation and the American College of Cardiology/American Heart Association (ACC/AHA) recommended practice guidelines to reduce the risk of CHD events [3, 4]. The association of several risk factors (RF) with CHD has led to development of a couple of CHD risk prediction models. These models incorporate information about age, sex, systolic blood pressure, cholesterol, smoking habits and diabetes mellitus to predict CHD risk. The quantification of coronary artery calcification (CAC) has been shown to allow better risk prediction of future CHD events then traditional risk prediction models [5–7]. Currently, the Multi Ethnic Study of Atherosclerosis (MESA) investigating group has developed a useful tool that includes CAC as well as established RFs to predict CHD risk [6].

The heritability of CHD has been estimated to be between 40 and 60% based on family, twin and genome-wide association studies (GWAS) [8–10]. Genetic risk scores based on single nucleotide polymorphisms (SNPs) that are associated with coronary artery disease (CAD) have been shown to improve the risk prediction of CHD [11–16]. Most of these genetic studies used Framingham or established RFs to estimate the CHD risk. A recent study from MESA could show an association between CAD genetic risk score and incident CHD only in men [11]. However, it has not been fully assessed i) whether the use of a CAD genetic risk score is superior to the measurement of CAC for CHD risk assessment and ii) whether the CHD risk assessment using a CAD genetic risk score differs between men and women.

## Methods

### Study population

Heinz Nixdorf Recall study is a population based cohort study consisting of 4814 participants, aged 45 to 75 years (50% women) at baseline. The study participants were randomly selected from the registration lists of the densely populated Ruhr metropolitan cities in Germany (residents of Essen, Bochum, and Mülheim an der Ruhr) between December 2000 and August 2003. The rationale and design of the study were described in detail previously [17]. The participants were re-invited for first and second follow-up examination taken place approximately 5 and 10 years after the baseline examinations. Participants with prior CHD (coronary artery bypass surgery and/or interventional revascularization, history of prior myocardial infraction and stroke) (*n* = 327) at baseline were excluded from the present study.

### Coronary heart disease

Incidental CHD was the primary end-points of our study. A pre-defined study criteria were used to clearly document the incidental CHD [18]. Hospital and nursing home records along with ECGs, laboratory values and pathology reports were collected for all the primary end-points [19]. Death certificates and interviews with general practitioners, relatives and eye witness were also obtained. Medical reports were further collected for all the reported end points [20]. All the documents were studied by an external end-point committee who were blinded for RF status. The end-point committee further classified the end points at separate regular meetings twice a year. CHD was defined as fatal and non-fatal myocardial infarction, stroke and coronary death. All the incident CHD that occurred between the baseline and third examination (*n* = 343, 8.5%) was included in the present study.

### Assessment of coronary artery calcification

Non-enhanced electron-beam scan (C-100 or C-150 scanner, GE Imatron, San Francisco, CA, USA), was used to assess CAC at baseline [17]. The prospective ECG-triggering was performed at 80% of the RR-interval. At an image acquisition time of 100 ms, contiguous 3 mm thick slices from the pulmonary bifurcation to the apex of the heart were then obtained in both the scans [21]. CAC was quantified using the methods of Agatston et al. [22]. The total CAC score was computed which comprised of all the calcified lesions in the coronary artery system. Virtuoso workstation (Siemens Medical Solutions, Forchheim, Germany) was used to perform the analyses. The results of CT scan were not disclosed to the study center or to the participants. We further categorized CAC into two groups i.e. CAC = 0 (absence of CAC) and CAC > 0 (presence of CAC).

### Cardiovascular risk factors

Cardiovascular RFs were recorded at baseline. As described previously smoking status (smokers (defined as current or past smokers) and non-smoker) was assessed in detail [23]. Current regular use of medication which included antihypertensive or lipid lowering medications was recorded in a standardized assessment of medications. We calculated the body mass index (BMI) as weight divided by height square (kg/m^2^). As described previously, resting blood pressure was measured using an automated oscillometric blood pressure device (Omron, HEM-705CP-E) with the participants seated. Of the three measurements the mean of the second and third value was calculated [24]. Serum triglycerides, low density lipoprotein (LDL)-cholesterol and high density lipoprotein (HDL)-cholesterol values were determined using the standardized enzymatic methods (ADVIA 1650, Siemens Medical Solutions, Erlangen, Germany). Diabetes was defined as either of 4 criteria: (1) participants reported a history of clinically diagnosed diabetes, (2) participants took glucose-lowering medications, (3) participants had fasting glucose levels (FPG) of greater than 125 mg/dL, or (4) participants had non-fasting glucose levels of 200 mg/dL or greater [25].

### Genotyping and genetic risk score

In the Heinz Nixdorf Recall study, 4371 participants were genotyped using Illumina GWAS chips (Omni1, OmniExpress, OmniExpress1, HumanCoreExome (v1.0 and v1.1); Illumina, San Diego, USA) and 4518 participants using Metabochip [26, 27]. Quality control (QC) was performed separately for all the chips at the subject level and then on SNPs before imputing each chip with IMPUTE v2.3.1 with reference data from 1000 Genomes Phase 1 release March 2012 for the Metabochip and 1000 Genomes Phase 3, release October 2014 for all the other chips [26, 28, 29]. At subject level, the QC involved sex-, ethnicity- and relatedness-checks. We excluded the participants if HET > 5 standard deviations of the mean, > 5% missing genotype data and outliers identified by principle component analysis. Thereafter, we excluded the SNPs with a minor allele frequency (MAF) < 1%, a missing genotype frequency > 5% or a deviation from Hardy–Weinberg Equilibrium (HWE) (*p* < 10^− 5^). GTOOL v0.7.5 (threshold ≥0.8) was next used to convert the imputed data into the PLINK ped format.

For this study, 70 SNPs were selected based on the published CAD GWAS (*p* ≤ 5 × 10^− 8^) to construct the genetic risk score [30–33] and recently published by Pechlivanis et al. [34]. The average weighted genetic risk score for each individual was constructed by using the risk estimate (transformed by natural log) from the published CAD GWAS and multiplying by the number of CAD risk alleles; these products were then summed up. The summed up product was then divided by the number of SNPs (*n* = 70). The allelic scoring routine in PLINK was used to calculate the genetic risk score [35]. The expected value based on the sample allele frequency was imputed, if the genotype in the score for a particular individual was missing. The mean (0.03) and standard deviation (SD; 0.003) of the study population were used to standardize the genetic risk score to have a mean of zero and unit variance. Genetic risk was then analyzed per-SD of the standardized genetic risk score. For our analyses we used 4041 participants having information on genetic risk score, sex, age and CAC at baseline.

### Statistical analysis

We calculated the Kaplan-Meier estimates of event-free survival probabilities in all the study participants as well as participants stratified by sex which was evaluated using a log-rank test of trend. The association of the genetic risk score with incident CHD was assessed with multivariable Cox proportional hazards regression to calculate the adjusted hazard ratios and corresponding 95% confidence intervals. The models were first adjusted for age and sex and the established RF adjusted model consisted of age, sex, systolic blood pressure, antihypertensive medication, smoking, LDL-cholesterol, HDL-cholesterol, lipid lowering medication, BMI, diabetes and CAC. The genetic risk score was analyzed as a continuous variable defined by per-SD of the standardized CAD genetic risk score and as ordinal scaled distribution to define genetic risk groups using quintiles (Q1-Q5). Q1 characterizes the low, Q2-Q4 the intermediate and Q5 the high genetic risk group.

The association between the genetic risk score and CAC was assessed in a logistic regression model. We excluded the participants with any missing data from the respective analysis.

Uno’s concordance statistics was used to evaluate the risk predictions [36]. Additionally, to find the best model to describe the relationship between genetic risk score and incident CHD we used Akaike’s information criterion (AIC) [37]. In our calculations, the lower the AIC value, the better was the model.

We controlled for multiple testing at 5% for our primary question relating the association of the genetic risk score per-SD with incident CHD in all, men and women study participants for the age and sex as well as RF plus CAC adjusted models. Accordingly, we corrected for 6 statistical tests that translate into *α*_*BF*_ = 0.008 using the Bonferroni procedure.

In order to test the hypothesis of a causal association between CAC and CHD, a Mendelian randomization analysis using CAD GRS as an instrumental variable was carried out [38]. In our analysis, genetically determined CAC (as predicted by the CAD GRS) was regressed against CHD. The inverse-variance weighted (IVW) method was used using the summary statistics (beta and standard error i.e., by scaling the natural logarithm of the OR) for the associations of the CAD GRS with CAC (exposure) and (beta and standard error i.e., by scaling the natural logarithm of the HR) CHD (outcome) from our study. The analyses were first carried out using all the study participants and then stratified by sex.

The continuous data are presented as mean ± SD or median (first quartile: q(25), third quartile: q(75)) if the distribution of data were substantially skewed. We performed the tests for group differences for the continuous data using the Student’s *t* test or the Mann-Whitney *U* test. Count data are presented as frequency and percentage. The difference in the group was evaluated by the χ2 or the Fisher exact test. The statistical analyses were done using SAS v.9.4.

## Results

### Study characteristics

The baseline characteristics of the Heinz Nixdorf Recall study participants are shown in Table 1. During a median follow up time of 11.6 ± 3.7 years, 343 (8.5%) participants experienced CHD events (219 men). The mean and standard deviation (SD) of the weighted genetic risk score in the Heinz Nixdorf Recall study participants was 0.03 ± 0.003. The weighted genetic risk score was statistically significantly (*p* = 0.006) higher in those with events (0.04 ± 0.003) than without events (0.03 ± 0.003). CAC (log(CAC + 1)) was statistically significantly (*p* < 0.0001) higher in those with events (median (Q1; Q3), 4.8 (2.7; 6.1)) than without events (2.3 (0; 4.5)) (Table 1). Also, the proportion of participants with presence of CAC (CAC > 0) were statistically significantly (*p* < 0.0001) higher in those with events (86.9%) than without events (65.9%). The amount of CAC increased with increase in the genetic risk group. Participants in the high genetic risk group showed the highest CAC values (log(CAC + 1)) (high genetic risk: 3.2 (0; 5.2), intermediate genetic risk: 2.5 (0; 4.7) and low genetic risk: 1.9 (0; 4.4)) (data not shown). Also, higher CAC was observed in men (high genetic risk: 4.5 (2.2; 5.8), intermediate genetic risk: 4.0 (1.7; 5.5) and low genetic risk: 3.6 (0.7; 5.0)) as well as in women (high genetic risk: 1.3 (0; 4.0), intermediate genetic risk: 0.9 (0; 3.5) and low genetic risk: 0.7 (0, 3.3)) belonging to the higher genetic risk group (data not shown). Kaplan-Meier curves for CHD events are shown in the Additional file Fig. 1 (a) and (b). An effect in men can be detected in the Kaplan-Meier survival curves (Additional file: Fig. 1 (b)), when the nonadjusted data were used.

**Table 1:** Characteristics of the Heinz Nixdorf Recall study population

	All Events/N 343/4041	Events N (%) 343 (8.5)	No Events N (%) 3693 (91.5)	*p*^c^
Age (years) ^a^	58.9 ± 7.6	63.5 ± 7.8	58.9 ± 7.6	< 0.0001
Men	1919 (47.5)	219 (63.9)	1700 (46.0)	< 0.0001
BMI (kg/m^2^)	27.7 ± 4.6	28.7 ± 4.2	27.72 ± 4.6	< 0.0001
Smoker	2291 (56.7)	211 (61.5)	2080 (56.3)	0.06
Diabetes	490 (12.1)	78 (22.7)	412 (11.1)	< 0.0001
Systolic blood pressure (mmHg)	131.8 ± 20.2	142.5 ± 23.6	131.8 ± 20.2	< 0.0001
Diastolic blood pressure (mmHg)	81.3 ± 10.7	83.8 ± 12.3	81.3 ± 10.7	0.0004
Use of antihypertensive medication	1263 (31.3)	163 (47.5)	1100 (29.8)	< 0.0001
log(CAC + 1) ^b^	2.3 (0; 4.5)	4.8 (2.7; 6.1)	2.3 (0; 4.5)	< 0.0001
CAC > 0	2734 (67.7)	298 (86.9)	2436 (65.9)	< 0.0001
Use of lipid lowering medication	360 (9.5)	39 (12.2)	321 (9.3)	0.11
LDL-cholesterol (mg/dL)	146.8 ± 36.3	147.9 ± 34.7	146.8 ± 36.3	0.40
HDL-cholesterol (mg/dL)	59.4 ± 17.1	55.2 ± 16.8	59.4 ± 17.1	< 0.0001
Triglycerides (mg/dL) ^b^	123.0 (89.0; 177.0)	141.0 (101.0; 203.0)	121.0 (88.0; 175.0)	< 0.0001
Total cholesterol ^a^	231.4 ± 38.7	232.2 ± 39.5	231.3 ± 38.6	0.73
Coronary artery disease GRS ^a^	0.03 ± 0.003	0.04 ± 0.003	0.03 ± 0.003	0.006

*LDL* low density lipoprotein, *HDL* high density lipoprotein, *CAC* coronary artery calcification, *GRS* genetic risk score. Data are given as number (percentage) unless otherwise indicated

^a^ Data are given as mean ± SD. ^b^ Data are given as median (Q1; Q3)

^c^*p* are for differences between CHD stratified groups using χ2 or Fisher exact test, t test or Mann-Whitney U test

### Genetic risk score and incident coronary heart disease

**Fig. 1:**
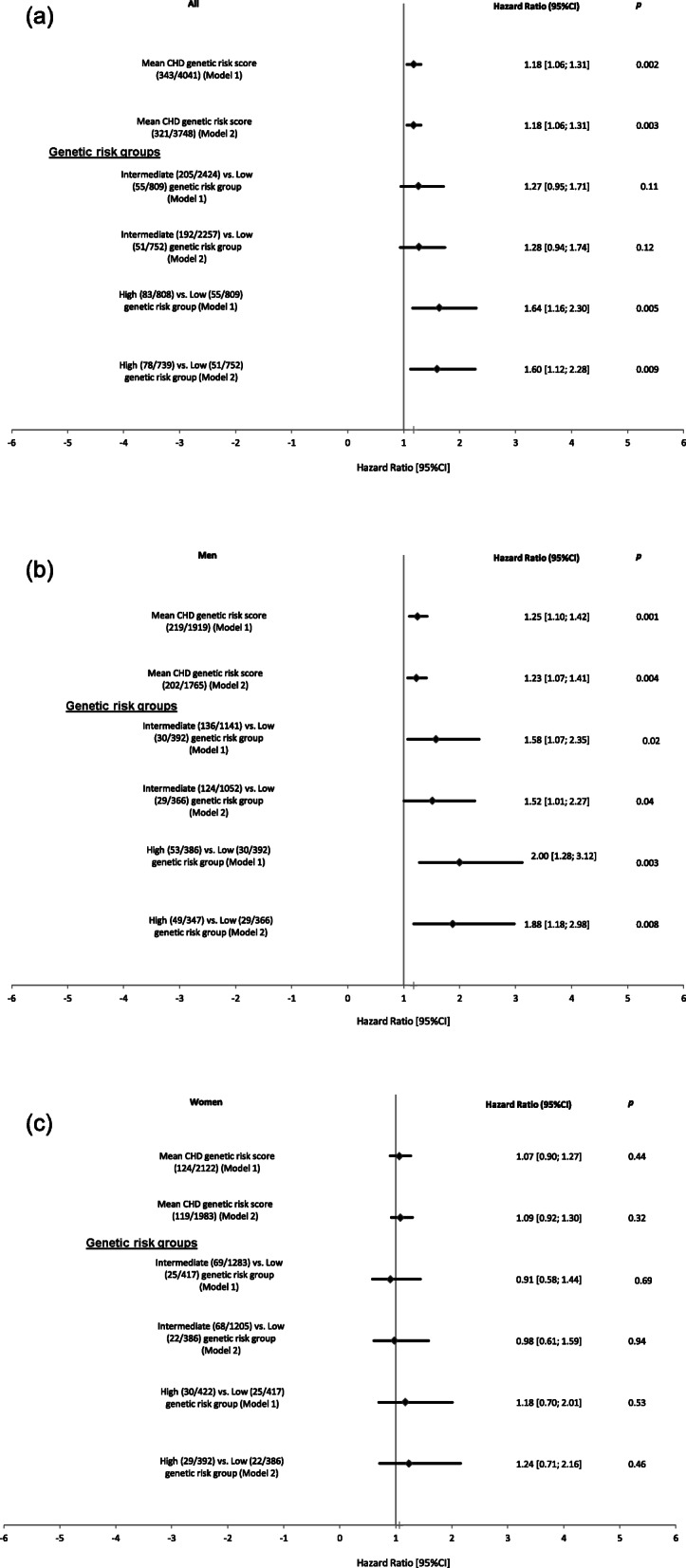
Effect of coronary artery disease genetic risk score with incident coronary heart disease. **a** all study participants, **b** men and **c** women. Model 1 is adjusted for age and sex. Model 2 is adjusted for age, sex, smoking, body mass index, diabetes, low density lipoprotein-cholesterol, high density lipoprotein-cholesterol, systolic blood pressure, use of antihypertensive and lipid lowering medication and coronary artery calcification. The numbers in parentheses are given as the (number of events/total number of participants). Additionally, in the sex stratified analyses we excluded the variable sex from the analysis

After adjusting for age and sex, the CAD genetic risk score was statistically significantly associated with incident CHD (hazard ratios (HR) =1.18 per-SD; 95% confidence interval [95% CI] [1.06; 1.31], *p* = 0.002) even after adjusting for multiple testing. Further adjusting for established RFs including systolic blood pressure, antihypertensive medication, smoking, LDL-cholesterol, HDL-cholesterol, lipid lowering medication, BMI, diabetes and CAC showed similar statistically significant effect (HR = 1.18 per-SD [1.06; 1.31], *p* = 0.003) (Fig. 1(a)). In the sex stratified analyses, men showed statistically significant higher risk for incident CHD (HR_men_ = 1.25 per-SD [1.10; 1.42], *p* = 0.001) with no statistically significant risk observed in women (HR_women_ = 1.07 per-SD [0.90; 1.27], *p* = 0.44) in the age adjusted analyses (Fig. 1 (b) and (c)).

The HR for incident CHD was statistically significantly higher in the high genetic risk group (high: 1.64 [1.16; 2.30], *p* = 0.005 and intermediate: 1.27 [0.95; 1.71], *p* = 0.11) compared to the low genetic risk group in the age and sex adjusted model. Adjusting for established RFs and CAC did not change the risk estimate (high: 1.60 [1.12; 2.28], *p* = 0.009) (Fig. 1(a)). Stratifying by sex, the risk was statistically significant for men in the high genetic risk group (HR = 1.88 [1.18; 2.98], *p* = 0.008) and borderline statistical significant for the intermediate genetic risk group (HR = 1.52 [1.01; 2.27], *p* = 0.04) when compared to the low genetic risk group after adjusting for established RFs and CAC (Fig. 1(b)). However, no statistically significant risk was observed for women (high: 1.24 [0.71; 2.16], *p* = 0.46 and intermediate: 0.98 [0.61; 1.59], *p* = 0.94) (Fig. 1(c)). Summary statistics for the association between the individual SNP and incident CHD in the Heinz Nixdorf Recall study participants is shown in the Additional file (Table 1).

We further did CAC stratified analyses (CAC = 0 and CAC > 0) and looked at the association of the genetic risk score with incident CHD in each stratum adjusting for established CHD RFs (Additional file: Fig. 2 (a-f)). The CAD genetic risk score was statistically significantly associated with incident CHD only in participants with presence of CAC (CAC > 0) (HR = 1.21 per-SD [1.08; 1.36], *p* = 0.001) (Additional file: Fig. 2(d)). Also, the high genetic risk group with higher CAC (CAC > 0 stratum) had statistically significantly higher risk for incident CHD (HR = 1.70 [1.16; 2.50], *p* = 0.007). In CAC > 0 stratum, statistically significant higher risk was observed only in men in the high genetic risk group (HR = 2.00 [1.23; 3.25], *p* = 0.005) and showed borderline statistical significance for the intermediate genetic risk group (HR = 1.52 [0.98; 2.34], *p* = 0.06) (Additional file: Fig. 2(e)). No statistically significant association was observed for women (Additional file: Fig. 2(f)).

### Genetic risk score and coronary artery calcification

Lastly, we looked at the association between the CAD genetic risk score and CAC. The CAD genetic risk score was statistically significantly associated with CAC. Per-SD increase in genetic risk score in the multivariable adjusted model for presence of CAC was OR: 1.19 [1.10; 1.29], *p* = 1.30 × 10^− 5^. Additionally the intermediate (OR: 1.35 [1.10; 1.64], *p* = 0.004) and higher (OR: 1.62 [1.26; 2.07], *p* = 0.0002) genetic risk groups showed statistically significant association with presence of CAC when compared with the low genetic risk group. In the sex stratified analyses, CAD genetic risk score showed statistically significantly stronger effects in men in the intermediate (OR: 1.90 [1.39; 2.60], *p* = 6.4 × 10^− 5^) and high (OR: 2.37 [1.56; 3.60], *p* = 4.9 × 10^− 5^) genetic risk groups when compared to the low genetic risk group. No statistically significant risk was observed for women in different genetic risk groups (Table 2).

**Table 2:** Association of coronary artery disease genetic risk score with coronary artery calcification in the Heinz Nixdorf Recall study

	Mean GRS OR [95% CI], *p*	Low	Intermediate OR [95% CI], *p*	High OR [95% CI], *p*
**All**				
N	4041	809	2424	808
Model 1	1.18 [1.10; 1.27], 6.37 × 10^−6^	Ref.	1.29 [1.07; 1.55], 0.007	1.57 [1.25; 1.96], 9.7 × 10^−5^
N	3748	752	2257	739
Model 2	1.19 [1.10; 1.29], 1.30 × 10^−5^		1.35 [1.10; 1.64], 0.004	1.62 [1.26; 2.07], 0.0002
**Men**				
N	1919	392	1141	386
Model 1	1.28 [1.13; 1.44], 5.60 × 10^−5^	Ref.	1.67 [1.25; 2.23], 0.0005	2.17 [1.48; 3.17], 7.0 × 10^− 5^
N	1765	366	1052	347
Model 2	1.29 [1.13; 1.47], 0.0001		1.90 [1.39; 2.60], 6.4 × 10^−5^	2.37 [1.56; 3.60], 4.9 × 10^− 5^
**Women**				
N	2122	417	1283	422
Model 1	1.13 [1.03; 1.23], 0.009	Ref.	1.09 [0.87; 1.38], 0.45	1.30 [0.99; 1.73], 0.06
N	1983	386	1205	392
Model 2	1.13 [1.03; 1.24], 0.01	Ref.	1.07 [0.83; 1.38], 0.59	1.28 [0.94; 1.76], 0.12

N: total number of participants in the analyses. *OR* odds ratio, *[95%CI]* 95%confidence interval, *Ref* reference. Model 1 is adjusted for age and sex. Model 2 is adjusted for age, sex, diabetes, body mass index, systolic blood pressure, smoking, antihypertensive medication, lipid lowering medication, low density lipoprotein-cholesterol and high density lipoprotein-cholesterol

### Discrimination

Comparing the model consisting of established RFs and CAC to the model including established RFs and genetic risk score did not reveal improvement of model (Δ Uno’s concordance statistics_All_ = 0.0042 ± 0.0045, *p* = 0.35) (Additional file: Fig. 3(a)). In the sex stratified analyses, men showed no improvement of the models (RF + CAC and RF + GRS: Δ Uno’s concordance statistics = − 0.0025 ± 0.0074, *p* = 0.74) (Additional file: Fig. 3(b)). Borderline significant improvement was observed in women when comparing models consisting of established RF and CAC to established RF and genetic risk score (Δ Uno’s concordance statistics = 0.0118 ± 0.0055, *p* = 0.03) (Additional file: Fig. 3(c)). However, it is to note that both the curves almost overlap each other (Additional file: Fig. 3(c)).

Subsequent AIC values suggested the model consisting of established RFs and CAC in all study participants (AIC value: 4952.851) to be superior compared to the model with established RFs and genetic risk score (AIC value: 4954.940). In sex stratified analyses, the model consisting of established RFs and genetic risk score (AIC value: 2856.279) is superior to the model consisting of established RFs and CAC in men (AIC value: 2859.856). However, the model consisting of established RFs and CAC (AIC value: 1673.447) was superior compared to the model with established RFs and genetic risk score in women (AIC value: 1678.255).

### Mendelian randomization, with coronary artery disease genetic risk score as the instrumental variable, to assess the causality of CAC for coronary heart disease

The Mendelian randomization analysis using IVW method showed that genetically predicted CAC is a causal risk factor for CHD in men and not in women, with an estimate of 0.90 for the presence of CAC (Estimate (95%CI), *p*: 0.90 [0.36; 1.44], *p* = 0.001) (Table 3).

**Table 3:** Causal estimates of the presence of coronary artery calcification on coronary heart disease from Mendelian Randomization analysis

	CHD		
	Causal estimate	95%CI	*P*
IVW (All study participants)	1.023	[0.38; 1.67]	0.002
IVW (Men)	0.90	[0.36; 1.44]	0.001
IVW (Women)	0.572	[−0.88; 2.03]	0.441

*IVW* inverse-variance weighted, *CHD* coronary heart disease

## Discussion

In the large population-based Heinz Nixdorf Recall study, we investigated the association between CAD genetic risk score and incident CHD. The important findings of our study are i) the CAD genetic risk score was associated with incident CHD, showing stronger effect in men with no effect observed in women, ii) the effect of genetic risk score did not alter even after adjusting for CAC, iii) the CAD genetic risk score was associated with incident CHD only in the group with presence of CAC, showing stronger effect in men and no effect in women and iv) CAD genetic risk score was associated with CAC with stronger effect observed in men.

Several large-scale CAD GWAS have led the discovery of novel SNPs [30–33], few of them are recently published and are incorporated in genetic risk score in our study [33]. Similar to previous studies, our study could illustrate that the CAD genetic risk score constructed using 70 SNPs is associated with incident CHD [11–16, 39]. This effect was more prominent in the higher genetic risk group and especially in men. These results are similar to the findings from a recently published study from MESA, where only men showed higher risk for CHD [11]. CAC, independent of traditional risk factors, is a well know predictor of CHD events [5–7]. CAC has its own genetic component and is highly heritable [27, 40, 41]. None of the previous studies has used CAC to find if the association between the CAD genetic risk score and incident CHD could be altered by CAC. The second finding of our study show that, even after adjusting for RFs and CAC the association between the genetic risk score and incident CHD remained significant. Moreover, in the CAC stratified analyses the association between the genetic risk score and CHD was observed only in group with presence of CAC (CAC > 0). Especially men belonging to the high genetic risk group were at increased risk of CHD. However, in an observational study from Erbel et al. both men and women belonging to CAC ≥ 400 group showed similar event rate for men (8.3%) and women (8.2%) and similar higher risk for CHD [20]. The non-significant association of the genetic risk score in women belonging to the presence of CAC group could be attributed to the different reclassification of CAC used in the present study i.e. CAC = 0 as absence and CAC > 0 as presence of CAC compared to the previous observational study which used several CAC score risk groups (i.e. low (1–99), intermediate (100–399) and high (≥400) risk groups) [20]. Reclassifying men and women based on CAC score into low, intermediate and high risk groups could have yielded different results in the present study. Although the CAD genetic risk score showed strong association with incident CHD in men, comparing the model consisting of established RFs and CAC to the model consisting of RFs and genetic risk score did not improve the risk prediction for CHD when assessed by Uno’s concordance. On other hand, risk assessment by Uno’s concordance showed positive association in women. These results indicate that model consisting of RFs and CAC is more predictive for women when compared to the model containing RFs and genetic risk score. However, minor but positive results were observed for men in the AIC analyses. Further larger studies are required to evaluate if the risk prediction for CHD could be improved by addition of genetic risk score to the established RFs in men. Furthermore, the results of our study also showed that the CAD genetic risk score was associated with presence of CAC. The results of the combined analyses i.e. using all the study participants are similar to the findings from other studies [42, 43]. However, the sex stratified results showed higher risk only in men. Furthermore, using a Mendelian randomization approach; we found that genetically determined CAC was causally associated with CHD in men.

The present study has several strengths and limitations. The strengths of the study are its longitudinal design, long follow-up time of 11.6 ± 3.7 years, the stringent predefined end-point criteria, the external end-point committee and availability of data on CAC and other CHD established RFs. Along with its strengths the study has its limitations. The greatest limitation of this study is its moderate sample size. Due to its moderate sample size, we could stratify our study population based on CAC score only in two groups (CAC = 0 and CAC > 0), however, it would have been more valuable to further stratify the presence of CAC group into low (1–99), intermediate (100–399) and high (≥400) risk groups [20]. Larger studies based on presence of CAC stratified analyses could help to find if the genetic risk score is superior to CAC in the risk assessment of CHD in each CAC stratified groups. Furthermore, due to moderate sample size we could not reclassify our study participants based on the recommendations from ACC/AHA Guidelines 2018 [4]. The recommendation suggest the assessment of CAC for treatment decision making in uncertain patients or patients at moderate risk i.e. in patients having atherosclerotic cardiovascular disease risk of 7.5–20%. Further larger studies are required to evaluate the prediction capabilities of the genetic risk score in this uncertain risk group. Also, the results of our study as well as the study from MESA showed similar non-significant association of the genetic risk score with CHD in women, these findings need further investigation. Two important points could be inferred from these findings i) there are other genetic variants for women which are yet to be identified i.e., by conducting sex stratified GWAS as well as by including chromosome X genetic variants in the GWAS and ii) role of endogenous sex hormones. Studies have shown that premenopausal women when compared to men of the same age have a lower incidence and prevalence of CVD [44, 45]. This sex differences favoring women, seems to disappear after menopause [46, 47]. Studies have indicated the contribution of reduced levels of ovarian hormones to the higher risk of CVD in women [46, 48–50]. A recent observational study from MESA showed that the post-menopausal women having higher testosterone levels, lower estradiol levels and higher testosterone/estradiol ratio had an elevated risk for CHD events [51]. Further larger studies in women are essential to find if the interaction between the sex hormones and the genetic risk score or genetic variants plays a role in the risk assessment of CHD in women. Although our results suggest that genetic risk assessment in men in the higher genetic risk groups could be useful, however additional larger studies stratified by sex, different CAC risk groups [20] as well as intermediate risk groups as recommended by ACC/AHA [4] are needed to evaluate the usefulness of genetic risk score for assessment of CHD in men.

## Conclusions

The findings of our study suggest that the CAD genetic risk score could be useful for CHD risk prediction, at least in men belonging to the higher genetic risk group, but it does not outbalance the value of the CT-based quantification of CAC which works independently on both men as well as women and allows better risk stratification in both the genders.

## Supplementary information

**Additional file 1.**

## Data Availability

Due to data security reasons i.e. the data contain potentially participant identifying information, the Heinz Nixdorf Recall study does not allow sharing data as a public use file. However, other authors/researchers are allowed to access data upon request, which is the same way the authors of the present paper obtained the data. Data requests can be addressed to recall@uk-essen.de.

## Abbreviations

CAD: Coronary artery disease
CHD: Coronary heart disease
CAC: coronary artery calcification
Q/q: Quintiles
RF: Risk factor
CT: Computer tomography
ACC/AHA: American College of Cardiology/American Heart Association
MESA: Multi Ethnic Study of Atherosclerosis
GWAS: genome-wide association studies
SNP: single nucleotide polymorphism
BMI: body mass index
LDL: low density lipoprotein
HDL: high density lipoprotein
AIC: Akaike’s information criterion
IVW: inverse-variance weighted
