# Supplementary material for: Risk prediction for coronary heart disease by a genetic risk score - results from the Heinz Nixdorf Recall study
Source: BMC Med Genet. 2020 Sep 10;21:178. doi: 10.1186/s12881-020-01113-y (PMC7487988; doi:10.1186/s12881-020-01113-y)
Supplement: Supplementary file 1 — Additional file 1. [file 12881_2020_1113_MOESM1_ESM.docx]

Additional file

**Regression Analysis Models**

Detailed descriptions of all the regression models used in this study are provided below. The models are described as formulas that were used in SAS.

Figure 1 (a-c)

Cox models

1. **All study participants**

Model 1:

(Time to first CHD event) ~ GRS per-SD + Age + Sex

Model 2:

(Time to first CHD event) ~ GRS per-SD + Age + Sex + Systolic blood pressure + Use of antihypertensives + Current smoker + LDL-cholesterol + HDL-cholesterol + Lipid lowering medication + BMI + Diabetes + Coronary artery calcification

Genetic risk groups

Model 1:

(Time to first CHD event) ~ Genetic risk groups + Age + Sex

Model 2:

(Time to first CHD event) ~ Genetic risk groups + Age + Sex + Systolic blood pressure + Use of antihypertensives + Current smoker + LDL-cholesterol + HDL-cholesterol + Lipid lowering medication + BMI + Diabetes + Coronary artery calcification

1. **Men**

Model 1:

(Time to first CHD event) ~ GRS per-SD + Age

Model 2:

(Time to first CHD event) ~ GRS per-SD + Age + Systolic blood pressure + Use of antihypertensives + Current smoker + LDL-cholesterol + HDL-cholesterol + Lipid lowering medication + BMI + Diabetes + Coronary artery calcification

Genetic risk groups

Model 1:

(Time to first CHD event) ~ Genetic risk groups + Age

Model 2:

(Time to first CHD event) ~ Genetic risk groups + Age + Systolic blood pressure + Use of antihypertensives + Current smoker + LDL-cholesterol + HDL-cholesterol + Lipid lowering medication + BMI + Diabetes + Coronary artery calcification

1. **Women**

Model 1:

(Time to first CHD event) ~ GRS per-SD + Age

Model 2:

(Time to first CHD event) ~ GRS per-SD + Age + Systolic blood pressure + Use of antihypertensives + Current smoker + LDL-cholesterol + HDL-cholesterol + Lipid lowering medication + BMI + Diabetes + Coronary artery calcification

Genetic risk groups

Model 1:

(Time to first CHD event) ~ Genetic risk groups + Age

Model 2:

(Time to first CHD event) ~ Genetic risk groups + Age + Systolic blood pressure + Use of antihypertensives + Current smoker + LDL-cholesterol + HDL-cholesterol + Lipid lowering medication + BMI + Diabetes + Coronary artery calcification

Logistic regression model to test the association of GRS with coronary artery calcification

**All study participants**

Model 1:

(Categorized CAC) ~ GRS per-SD + Age + Sex

Model 2:

(Categorized CAC) ~ GRS per-SD + Age + Sex + Systolic blood pressure + Use of antihypertensives + Current smoker + LDL-cholesterol + HDL-cholesterol + Lipid lowering medication + BMI + Diabetes

Genetic risk groups

Model 1:

(Categorized CAC) ~ Genetic risk groups + Age + Sex

Model 2:

(Categorized CAC) ~ Genetic risk groups + Age + Sex + Systolic blood pressure + Use of antihypertensives + Current smoker + LDL-cholesterol + HDL-cholesterol + Lipid lowering medication + BMI + Diabetes

**Men**

Model 1:

(Categorized CAC) ~ GRS per-SD + Age

Model 2:

(Categorized CAC) ~ GRS per-SD + Age + Systolic blood pressure + Use of antihypertensives + Current smoker + LDL-cholesterol + HDL-cholesterol + Lipid lowering medication + BMI + Diabetes

Genetic risk groups

Model 1:

(Categorized CAC) ~ Genetic risk groups + Age

Model 2:

(Categorized CAC) ~ Genetic risk groups + Age + Systolic blood pressure + Use of antihypertensives + Current smoker + LDL-cholesterol + HDL-cholesterol + Lipid lowering medication + BMI + Diabetes

**Women**

Model 1:

(Categorized CAC) ~ GRS per-SD + Age

Model 2:

(Categorized CAC) ~ GRS per-SD + Age + Systolic blood pressure + Use of antihypertensives + Current smoker + LDL-cholesterol + HDL-cholesterol + Lipid lowering medication + BMI + Diabetes

Genetic risk groups

Model 1:

(Categorized CAC) ~ Genetic risk groups + Age

Model 2:

(Categorized CAC) ~ Genetic risk groups + Age + Systolic blood pressure + Use of antihypertensives + Current smoker + LDL-cholesterol + HDL-cholesterol + Lipid lowering medication + BMI + Diabetes

Additional file Table 1

Cox models

(Time to first CHD event) ~ SNP + Age + Sex

Additional file Fig 2 (a-f)

Cox models

1. **All study participants with CAC=0 stratum**

(Time to first CHD event) ~ GRS per-SD + Age + Sex + Systolic blood pressure + Use of antihypertensives + Current smoker + LDL-cholesterol + HDL-cholesterol + Lipid lowering medication + BMI + Diabetes + Coronary artery calcification

Genetic risk groups

(Time to first CHD event) ~ Genetic risk groups + Age + Sex + Systolic blood pressure + Use of antihypertensives + Current smoker + LDL-cholesterol + HDL-cholesterol + Lipid lowering medication + BMI + Diabetes + Coronary artery calcification

1. **Men with CAC=0 stratum**

(Time to first CHD event) ~ GRS per-SD + Age + Systolic blood pressure + Use of antihypertensives + Current smoker + LDL-cholesterol + HDL-cholesterol + Lipid lowering medication + BMI + Diabetes + Coronary artery calcification

Genetic risk groups

(Time to first CHD event) ~ Genetic risk groups + Age + Systolic blood pressure + Use of antihypertensives + Current smoker + LDL-cholesterol + HDL-cholesterol + Lipid lowering medication + BMI + Diabetes + Coronary artery calcification

1. **Women with CAC=0 stratum**

(Time to first CHD event) ~ GRS per-SD + Age + Systolic blood pressure + Use of antihypertensives + Current smoker + LDL-cholesterol + HDL-cholesterol + Lipid lowering medication + BMI + Diabetes + Coronary artery calcification

Genetic risk groups

(Time to first CHD event) ~ Genetic risk groups + Age + Systolic blood pressure + Use of antihypertensives + Current smoker + LDL-cholesterol + HDL-cholesterol + Lipid lowering medication + BMI + Diabetes + Coronary artery calcification

1. **All study participants with CAC>0 stratum**

(Time to first CHD event) ~ GRS per-SD + Age + Sex + Systolic blood pressure + Use of antihypertensives + Current smoker + LDL-cholesterol + HDL-cholesterol + Lipid lowering medication + BMI + Diabetes + Coronary artery calcification

Genetic risk groups

(Time to first CHD event) ~ Genetic risk groups + Age + Sex + Systolic blood pressure + Use of antihypertensives + Current smoker + LDL-cholesterol + HDL-cholesterol + Lipid lowering medication + BMI + Diabetes + Coronary artery calcification

1. **Men with CAC>0 stratum**

(Time to first CHD event) ~ GRS per-SD + Age + Systolic blood pressure + Use of antihypertensives + Current smoker + LDL-cholesterol + HDL-cholesterol + Lipid lowering medication + BMI + Diabetes + Coronary artery calcification

Genetic risk groups

(Time to first CHD event) ~ Genetic risk groups + Age + Systolic blood pressure + Use of antihypertensives + Current smoker + LDL-cholesterol + HDL-cholesterol + Lipid lowering medication + BMI + Diabetes + Coronary artery calcification

1. **Women with CAC>0 stratum**

(Time to first CHD event) ~ GRS per-SD + Age + Systolic blood pressure + Use of antihypertensives + Current smoker + LDL-cholesterol + HDL-cholesterol + Lipid lowering medication + BMI + Diabetes + Coronary artery calcification

Genetic risk groups

(Time to first CHD event) ~ Genetic risk groups + Age + Systolic blood pressure + Use of antihypertensives + Current smoker + LDL-cholesterol + HDL-cholesterol + Lipid lowering medication + BMI + Diabetes + Coronary artery calcification

Additional file Fig 3 (a-c)

Cox models

1. **All study participants with Risk factors + Coronary artery calcification**

(Time to first CHD event) ~ Age + Sex + Systolic blood pressure + Use of antihypertensives + Current smoker + LDL-cholesterol + HDL-cholesterol + Lipid lowering medication + BMI + Diabetes + Coronary artery calcification

**All study participants with Risk factors + GRS per-SD**

(Time to first CHD event) ~ GRS per-SD + Age + Sex + Systolic blood pressure + Use of antihypertensives + Current smoker + LDL-cholesterol + HDL-cholesterol + Lipid lowering medication + BMI + Diabetes

1. **Men with Risk factors + Coronary artery calcification**

(Time to first CHD event) ~ Age + Systolic blood pressure + Use of antihypertensives + Current smoker + LDL-cholesterol + HDL-cholesterol + Lipid lowering medication + BMI + Diabetes + Coronary artery calcification

**Men with Risk factors + GRS per-SD**

(Time to first CHD event) ~ GRS per-SD + Age + Systolic blood pressure + Use of antihypertensives + Current smoker + LDL-cholesterol + HDL-cholesterol + Lipid lowering medication + BMI + Diabetes

1. **Women with Risk factors + Coronary artery calcification**

(Time to first CHD event) ~ Age + Systolic blood pressure + Use of antihypertensives + Current smoker + LDL-cholesterol + HDL-cholesterol + Lipid lowering medication + BMI + Diabetes + Coronary artery calcification

**Women with Risk factors + GRS per-SD**

(Time to first CHD event) ~ GRS per-SD + Age + Systolic blood pressure + Use of antihypertensives + Current smoker + LDL-cholesterol + HDL-cholesterol + Lipid lowering medication + BMI + Diabetes

**Fig 1.** Kaplan-Meier curves for coronary heart disease events in a) all study participants and b) stratified by sex. *P*-value from log-rank test of trend.

(a)


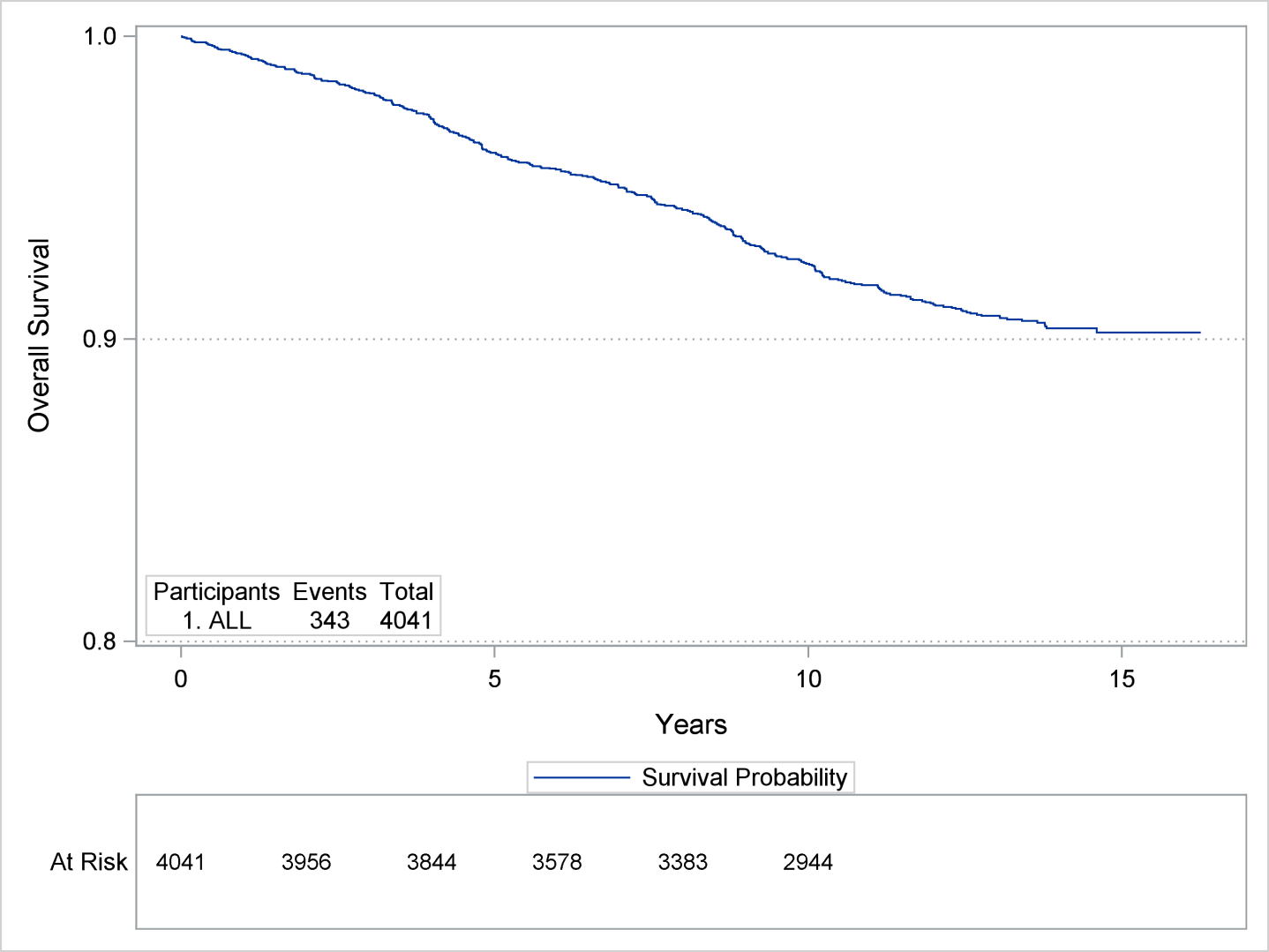


(b)


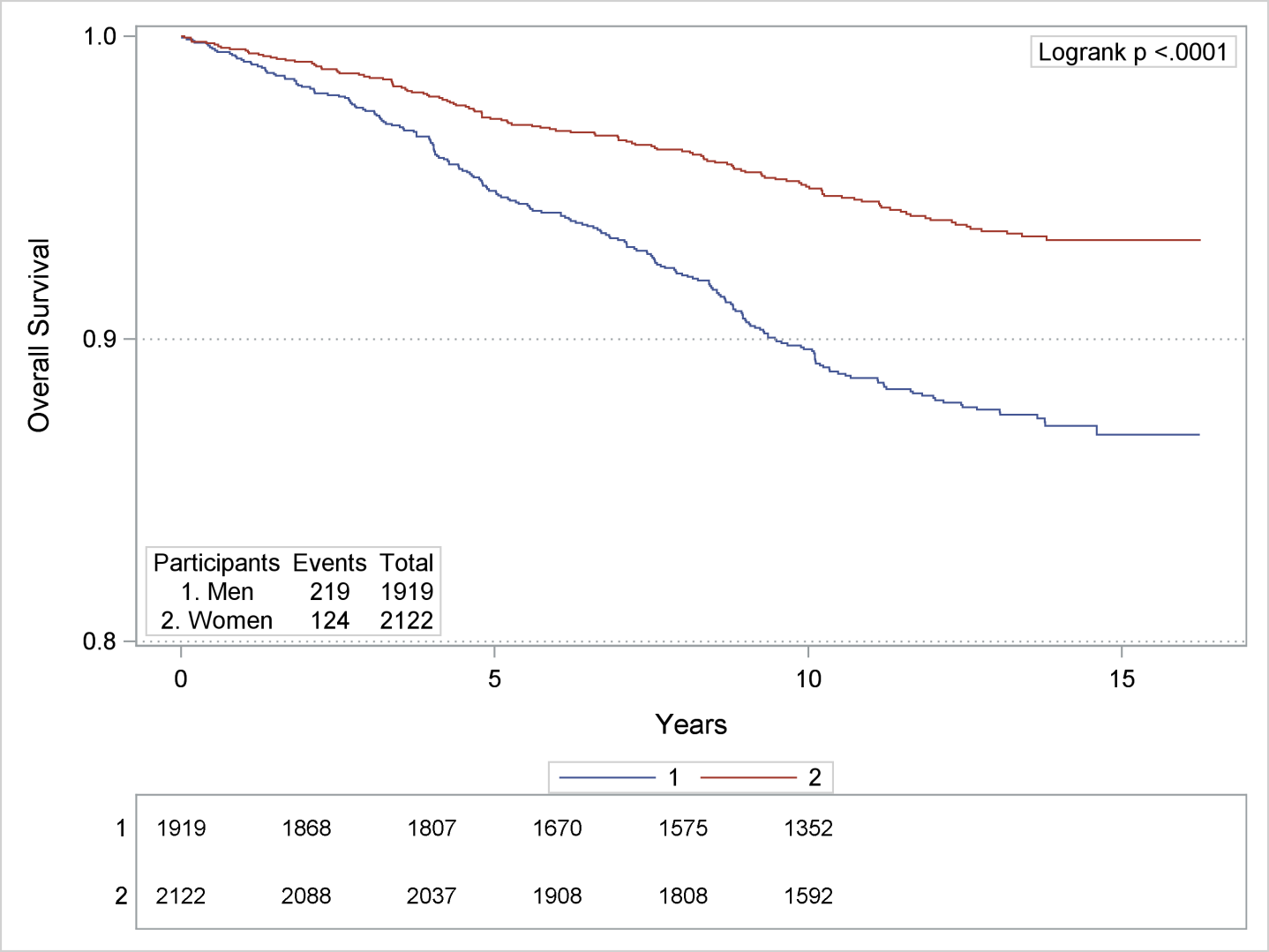


Table 1. Association of coronary artery disease associated SNPs with incident coronary heart disease in the Heinz Nixdorf Recall study

| CHR | SNP | BP | CA | NCA | CAF | HR [95%CI], P |
| --- | --- | --- | --- | --- | --- | --- |
| **1** | rs11206510 | 55496039 | T | C | 0.81 | 1.01 [0.83; 1.22], 0.93 |
| **1** | rs17114036 | 56962821 | A | G | 0.92 | 1.07 [0.81; 1.42], 0.22 |
| **1** | rs599839 | 109822166 | A | G | 0.77 | 1.14 [0.95;1.38], 0.15 |
| **1** | rs4845625 | 154422067 | T | C | 0.44 | 1.03 [0.88; 1.20], 0.72 |
| **1** | rs17464857 | 222762709 | T | G | 0.85 | 1.00 [0.82; 1.23], 0.98 |
| **1** | rs17465637 | 222823529 | C | A | 0.74 | 1.00 [0.84; 1.18], 0.97 |
| **2** | rs515135 | 21286057 | C | T | 0.82 | 0.96 [0.79; 1.17], 0.71 |
| **2** | rs6544713 | 44073881 | T | C | 0.31 | 0.97 [0.83; 1.15], 0.75 |
| **2** | rs1561198 | 85809989 | T | C | 0.45 | 1.08 [0.93; 1.26], 0.30 |
| **2** | rs2252641 | 145801461 | C | T | 0.44 | 1.01 [0.87; 1.17], 0.91 |
| **2** | rs6725887 | 203745885 | C | T | 0.13 | 0.86 [0.68; 1.09], 0.20 |
| **2** | rs1801251 | 233633460 | A | G | 0.33 | 1.15 [0.98; 1.35], 0.10 |
| **3** | rs9818870 | 138122122 | T | C | 0.17 | 1.18 [0.98; 1.42], 0.09 |
| **4** | rs17087335 | 57838583 | T | G | 0.20 | 0.87 [0.71; 1.05], 0.15 |
| **4** | rs1878406 | 148393664 | T | C | 0.14 | 1.22 [0.99; 1.49], 0.06 |
| **4** | rs7692387 | 156635309 | G | A | 0.80 | 1.04 [0.86; 1.25], 0.71 |
| **5** | rs273909 | 131667353 | G | A | 0.12 | 0.99 [0.79; 1.25], 0.93 |
| **6** | rs12526453 | 12927544 | C | G | 0.65 | 1.13 [0.96; 1.32], 0.15 |
| **6** | rs3130683 | 31888367 | T | C | 0.86 | 1.15 [0.91; 1.45], 0.26 |
| **6** | rs17609940 | 35034800 | G | C | 0.81 | 1.19 [0.96; 1.46], 0.11 |
| **6** | rs10947789 | 39174922 | T | C | 0.75 | 1.01 [0.85; 1.20], 0.89 |
| **6** | rs12190287 | 134214525 | C | G | 0.64 | 1.02 [0.85; 1.20], 0.89 |
| **6** | rs2048327 | 160863532 | C | T | 0.35 | 0.95 [0.81; 1.11], 0.52 |
| **6** | rs3798220 | 160961137 | C | T | 0.02 | 1.00 [0.54; 1.81], 0.98 |
| **6** | rs4252120 | 161143608 | T | C | 0.70 | 0.86 [0.73; 1.01], 0.06 |
| **7** | rs2023938 | 19036775 | C | T | 0.09 | 0.97 [0.75; 1.27], 0.85 |
| **7** | rs12539895 | 107091849 | A | C | 0.20 | 0.90 [0.74; 1.09], 0.26 |
| **7** | rs11556924 | 129663496 | C | T | 0.61 | 1.22 [1.04;1.43], 0.02 |
| **7** | rs3918226 | 150690176 | T | C | 0.09 | 1.00 [0.75; 1.32], 0.97 |
| **8** | rs264 | 19813180 | G | A | 0.86 | 1.00 [0.80; 1.24], 0.97 |
| **8** | rs2954029 | 126490972 | A | T | 0.51 | 0.97 [0.83; 1.13], 0.68 |
| **9** | rs3217992 | 22003223 | T | C | 0.38 | 1.09 [0.93; 1.27], 0.28 |
| **9** | rs1333049 | 22125503 | C | G | 0.46 | 1.10 [0.94; 1.28], 0.22 |
| **9** | rs579459 | 136154168 | C | T | 0.24 | 1.02 [0.85; 1.21], 0.90 |
| **10** | rs2505083 | 30335122 | C | T | 0.44 | 1.02 [0.88; 1.18], 0.82 |
| **10** | rs2047009 | 44539913 | G | T | 0.51 | 1.16 [1.00; 1.35], 0.05 |
| **10** | rs501120 | 44753867 | T | C | 0.86 | 1.01 [0.81; 1.26], 0.92 |
| **10** | rs11203042 | 90989109 | T | C | 0.42 | 0.98 [0.84; 1.14], 0.77 |
| **10** | rs2246833 | 91005854 | T | C | 0.32 | 1.07 [0.92;1.26], 0.38 |
| **10** | rs12413409 | 104719096 | G | A | 0.9 | 1.00 [0.78; 1.29], 0.98 |
| **11** | rs10840293 | 9751196 | A | G | 0.57 | 1.00 [0.85; 1.17], 0.96 |
| **11** | rs974819 | 103660567 | T | C | 0.28 | 1.08 [0.91; 1.27], 0.39 |
| **11** | rs11042937 | 10745394 | T | G | 0.46 | 0.86 [0.74; 1.00], 0.06 |
| **11** | rs9326246 | 116611733 | C | G | 0.07 | 1.06 [0.79; 1.42], 0.70 |
| **11** | rs964184 | 116648917 | G | C | 0.14 | 1.10 [0.88; 1.36], 0.42 |
| **12** | rs11172113 | 57527283 | C | T | 0.40 | 1.06 [0.91; 1.23], 0.46 |
| **12** | rs3184504 | 111884608 | T | C | 0.51 | 1.03 [0.87; 1.18], 0.87 |
| **12** | rs11830157 | 118265441 | G | T | 0.41 | 0.97 [0.82; 1.15], 0.72 |
| **12** | rs11057830 | 125307053 | A | G | 0.14 | 1.00 [0.81; 1.25], 0.97 |
| **13** | rs9319428 | 28973621 | A | G | 0.31 | 1.05 [0.90; 1.24], 0.53 |
| **13** | rs4773144 | 110960712 | G | A | 0.44 | 0.94 [0.81; 1.10], 0.43 |
| **13** | rs9515203 | 111049623 | T | C | 0.74 | 0.98 [0.83; 1.17], 0.85 |
| **14** | rs2895811 | 100133942 | C | T | 0.43 | 1.18 [1.01; 1.37], 0.04 |
| **15** | rs56062135 | 67455630 | C | T | 0.77 | 1.18 [0.98; 1.43], 0.08 |
| **15** | rs3825807 | 79089111 | A | G | 0.55 | 1.12 [0.96; 1.30], 0.16 |
| **15** | rs7173743 | 79141784 | T | C | 0.55 | 1.13 [0.97; 1.32], 0.11 |
| **15** | rs8042271 | 89574218 | A | G | 0.05 | 1.20 [0.80; 1.78], 0.38 |
| **15** | rs17514846 | 91416550 | A | C | 0.48 | 1.09 [0.94; 1.27], 0.25 |
| **16** | rs1800775 | 56995236 | C | A | 0.52 | 0.89 [0.77; 1.04], 0.13 |
| **17** | rs216172 | 2126504 | C | G | 0.34 | 1.00 [0.85; 1.18], 0.99 |
| **17** | rs12936587 | 17543722 | G | A | 0.55 | 0.97 [0.84; 1.13], 0.73 |
| **17** | rs46522 | 46988597 | C | T | 0.46 | 0.88 [0.75; 1.03], 0.11 |
| **17** | rs7212798 | 59013488 | C | T | 0.16 | 1.15 [0.94; 1.40], 0.18 |
| **18** | rs663129 | 57838401 | A | G | 0.24 | 1.11 [0.94;1.32], 0.23 |
| **19** | rs1122608 | 11163601 | G | T | 0.75 | 1.05 [0.89; 1.25], 0.63 |
| **19** | rs12976411 | 32882020 | A | T | 0.97 | 0.81 [0.52; 1.27], 0.35 |
| **19** | rs2075650 | 45395619 | G | A | 0.15 | 1.09 [0.89;1.33], 0.43 |
| **19** | rs445925 | 45415640 | G | A | 0.88 | 0.99 [0.78; 1.24], 0.90 |
| **21** | rs9982601 | 35599128 | T | C | 0.14 | 1.01 [0.82; 1.26], 0.91 |
| **22** | rs180803 | 24658858 | A | C | 0.99 | 1.65 [0.62; 4.41], 0.32 |

CHR: chromosome, BP: base position (hgBuild37), CA: coded allele, NCA: non coded allele, CAF: coded allele frequency, HR: hazard ratio, 95%CI: 95% confidence interval. The models are adjusted for age and sex.

**Fig.2.** Effect of coronary artery disease genetic risk score with incident coronary heart disease, stratified by coronary artery calcification. Fig 2(a-c) includes study participants having CAC=0: 2(a) all study participants, 2(b) men and 2(c) women. Fig 2(d-f) includes study participants having CAC>0: 2(d) all study participants, 2(e) men and 2(f) women. The models are adjusted for age, sex, smoking status, body mass index, diabetes, low density lipoprotein-cholesterol, high density lipoprotein-cholesterol, systolic blood pressure, use of antihypertensive and lipid lowering medication. The numbers in parentheses are given as the (number of events/total number of participants). Additionally, in the sex stratified analyses we excluded the variable sex from the analysis.


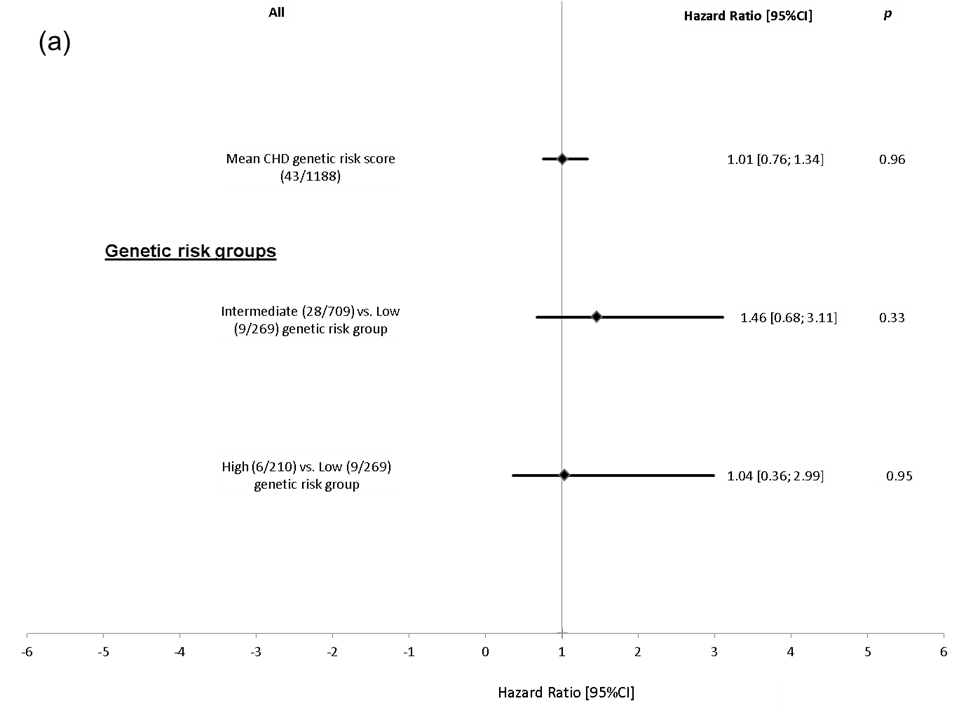


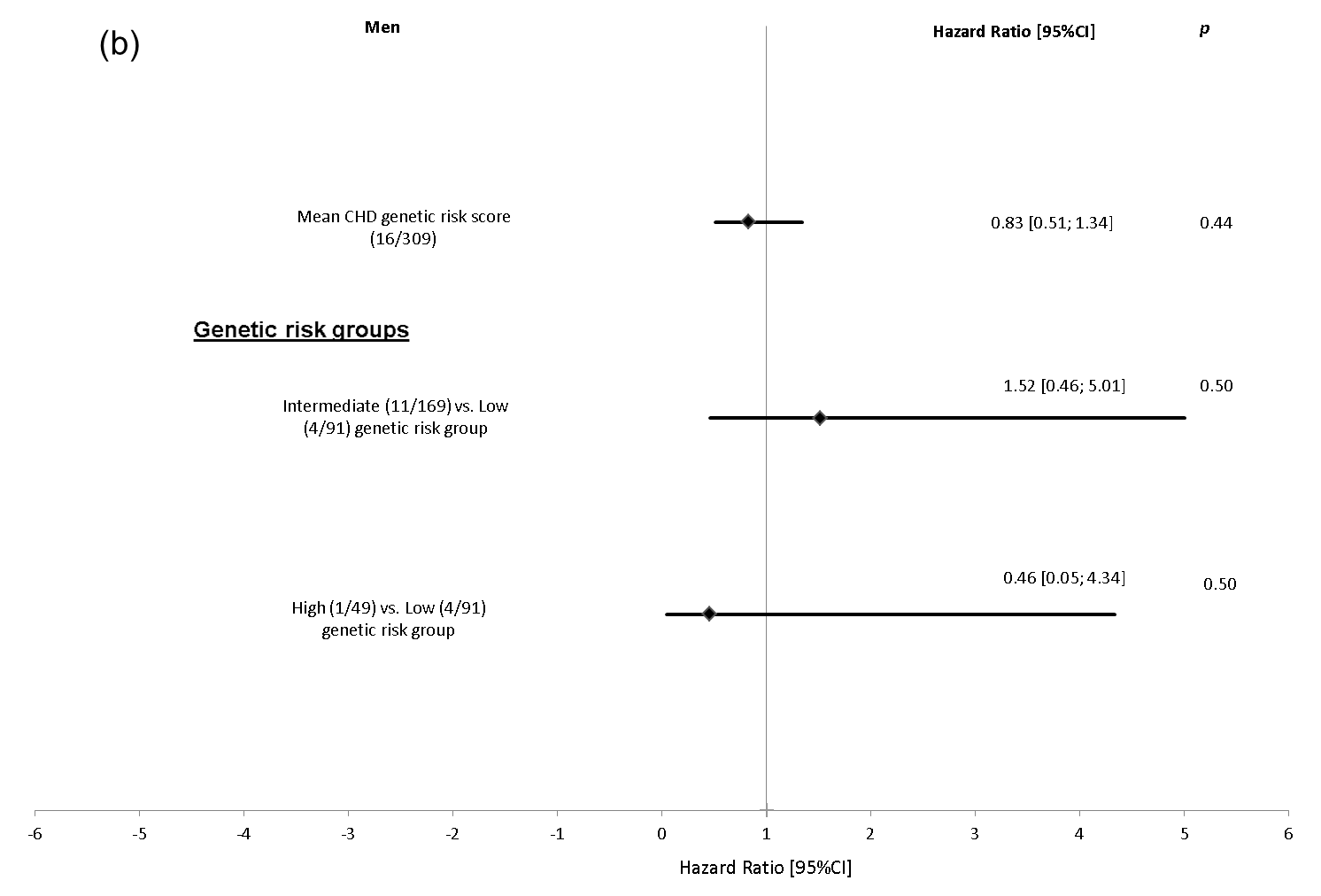


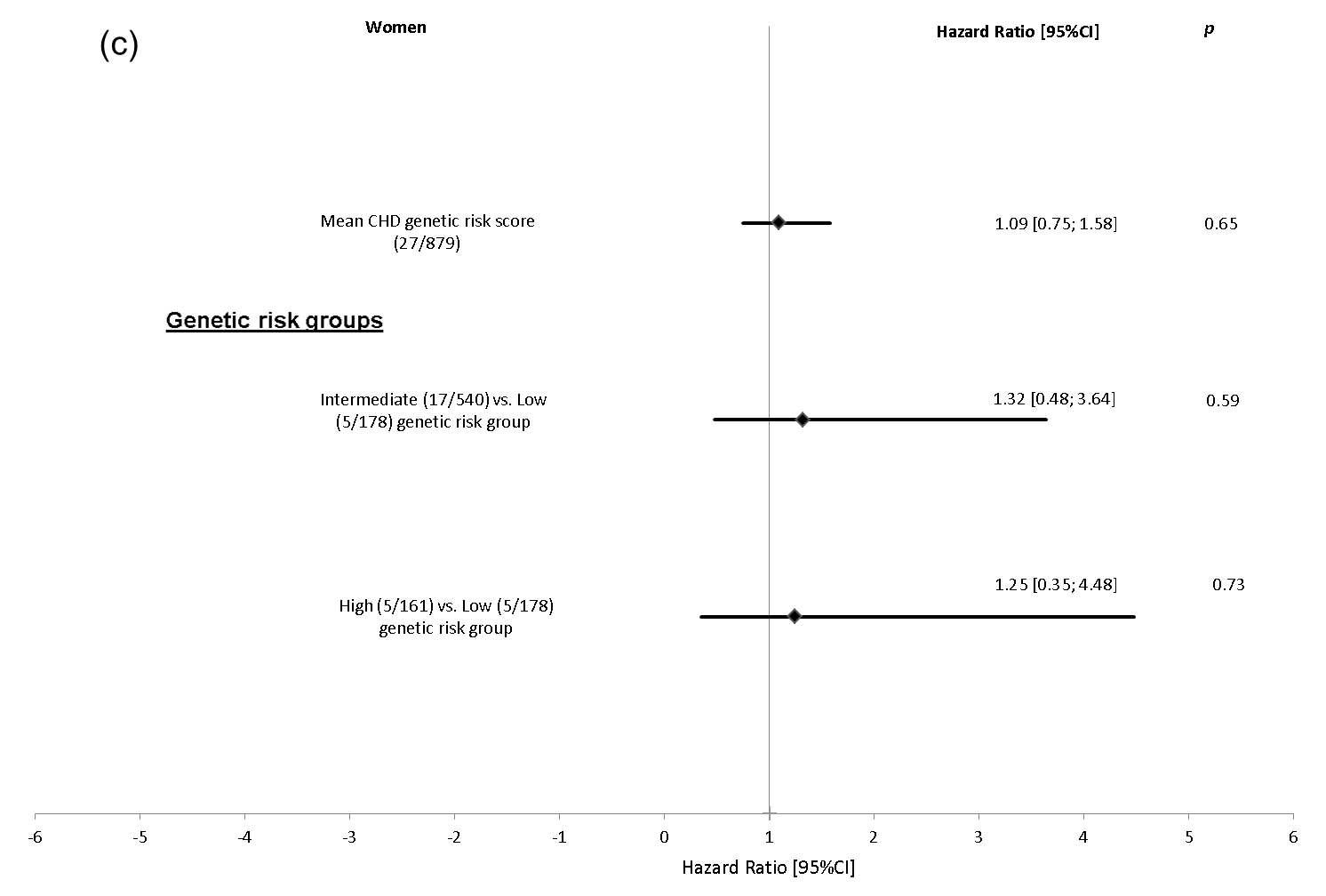


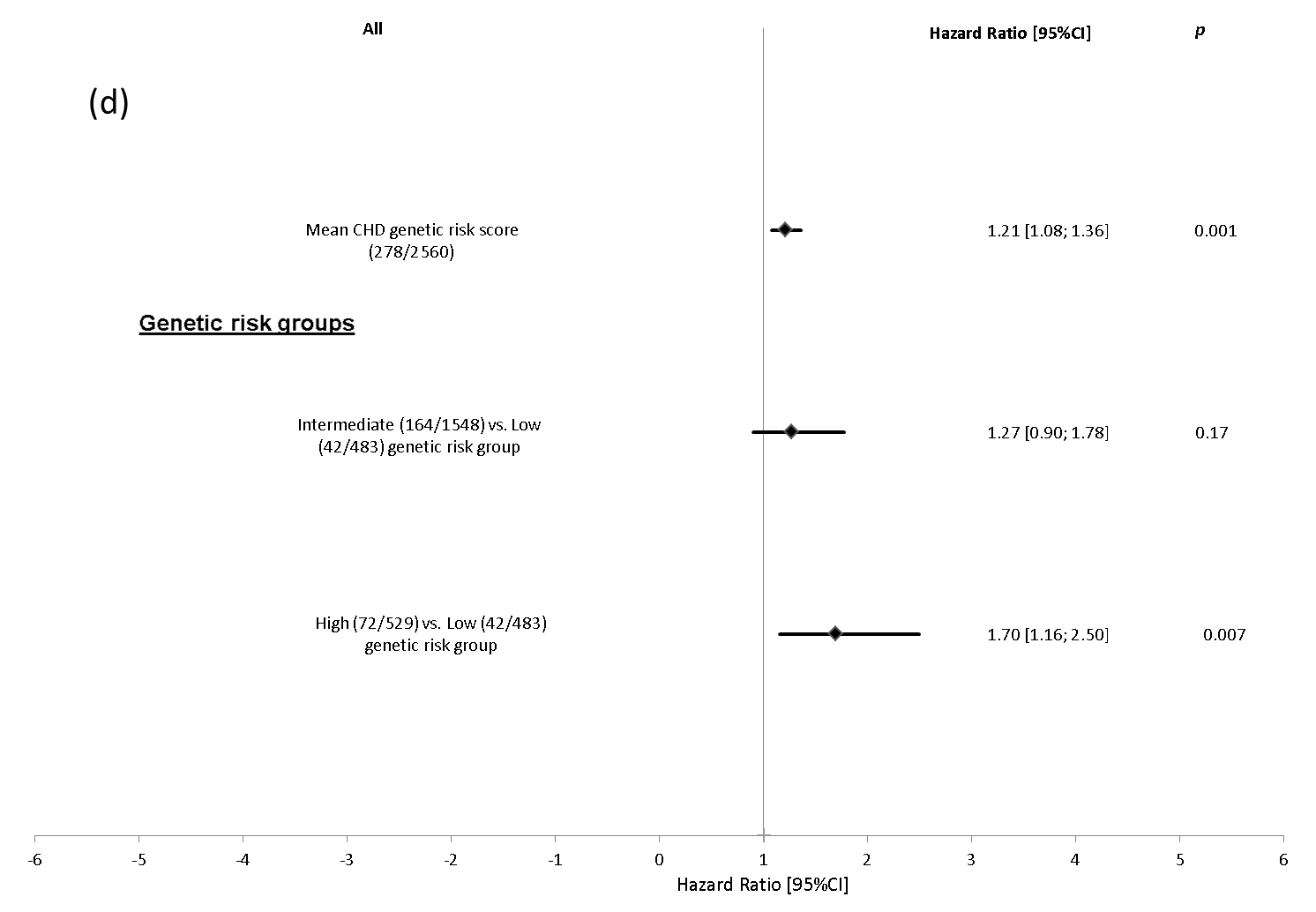


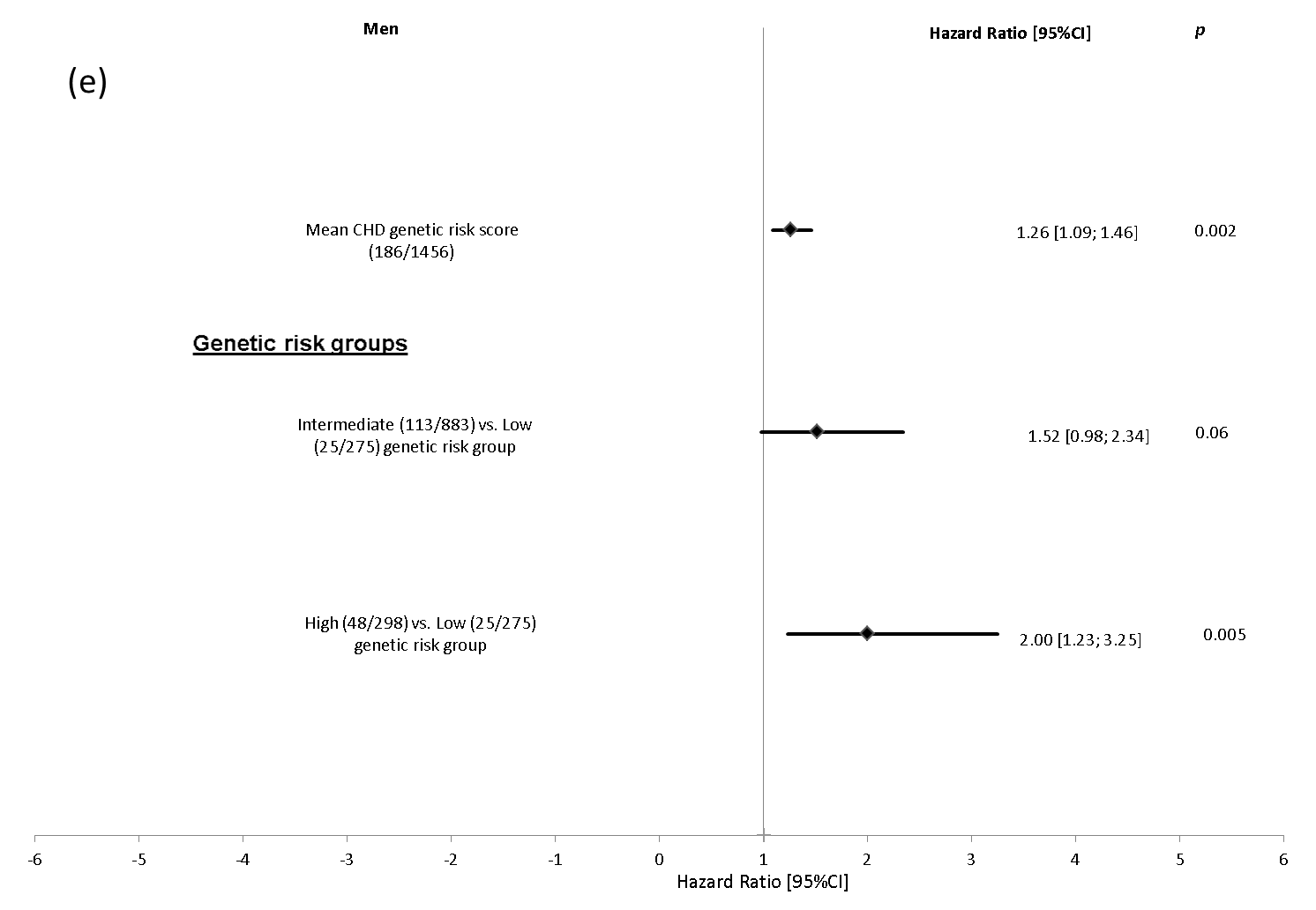


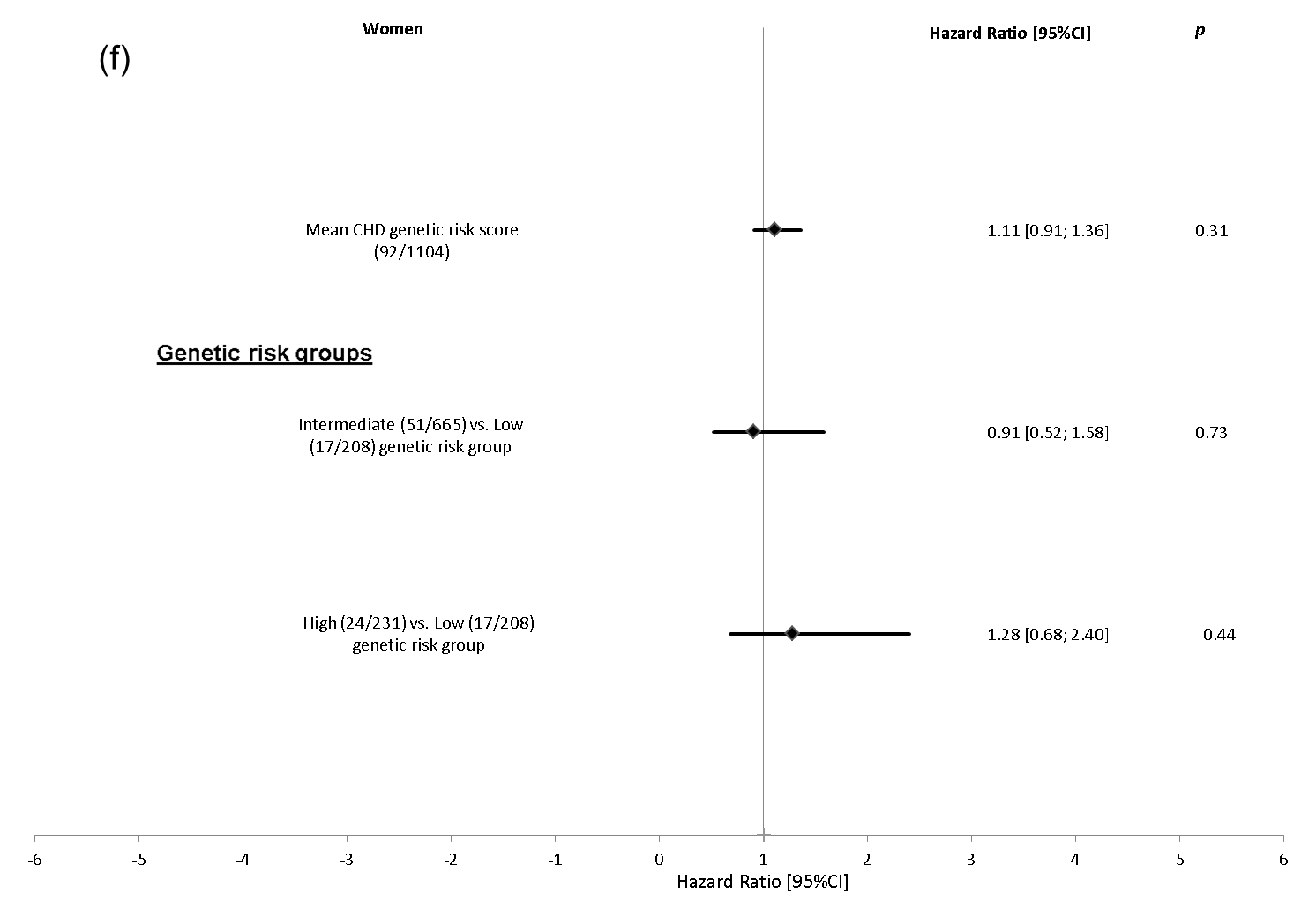


Fig 3: ROC curves illustrates the discrimination properties of the two models i) risk factors and CAC and ii) risk factors and genetic risk score for (a) all study participants, (b) men and (c) women. Uno’s Concordance Statistic are given as Estimate±Standard Error


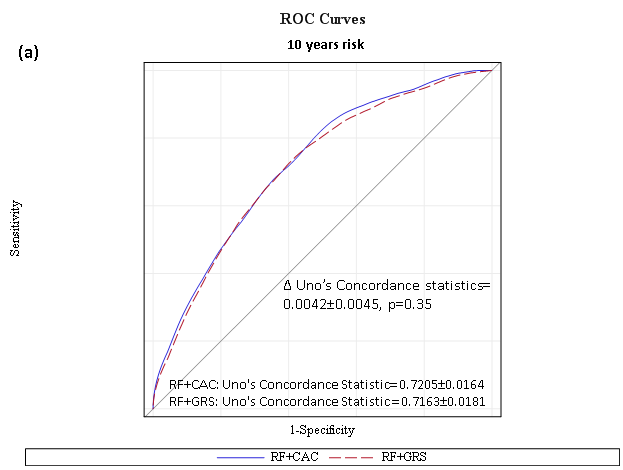


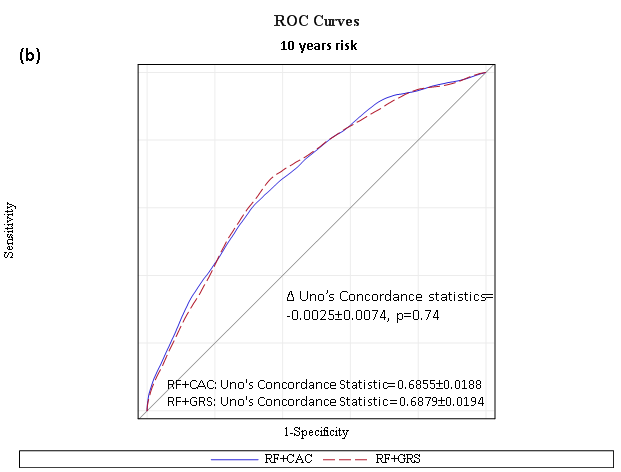


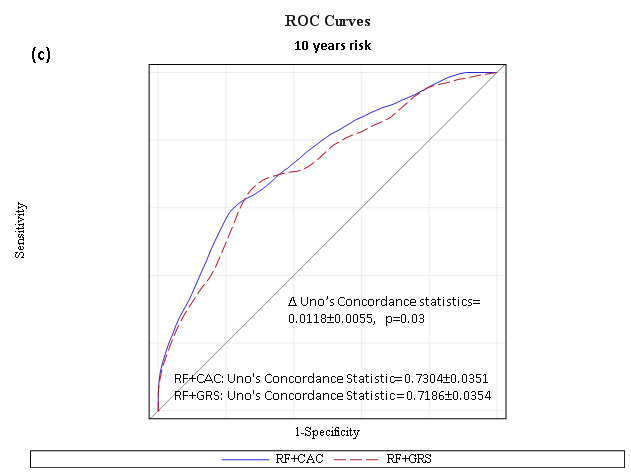


Risk factors: RF, coronary artery calcification: CAC and genetic risk score: GRS. The models are adjusted for following risk factors: age, sex, smoking status, body mass index, diabetes, low density lipoprotein-cholesterol, high density lipoprotein-cholesterol, systolic blood pressure, use of antihypertensive and lipid lowering medication.
